# Resources and Power Efficient FPGA Accelerators for Real-Time Image Classification

**DOI:** 10.3390/jimaging8040114

**Published:** 2022-04-15

**Authors:** Angelos Kyriakos, Elissaios-Alexios Papatheofanous, Charalampos Bezaitis, Dionysios Reisis

**Affiliations:** Electronics Laboratory, Faculty of Physics, National and Kapodistrian University of Athens, 15772 Athens, Greece; akyriakos@phys.uoa.gr (A.K.); eapapatheo@phys.uoa.gr (E.-A.P.); bezaitisc@phys.uoa.gr (C.B.)

**Keywords:** image processing, CNN, accelerator, FPGA, vessel detection

## Abstract

A plethora of image and video-related applications involve complex processes that impose the need for hardware accelerators to achieve real-time performance. Among these, notable applications include the Machine Learning (ML) tasks using Convolutional Neural Networks (CNNs) that detect objects in image frames. Aiming at contributing to the CNN accelerator solutions, the current paper focuses on the design of Field-Programmable Gate Arrays (FPGAs) for CNNs of limited feature space to improve performance, power consumption and resource utilization. The proposed design approach targets the designs that can utilize the logic and memory resources of a single FPGA device and benefit mainly the edge, mobile and on-board satellite (OBC) computing; especially their image-processing- related applications. This work exploits the proposed approach to develop an FPGA accelerator for vessel detection on a Xilinx Virtex 7 XC7VX485T FPGA device (Advanced Micro Devices, Inc, Santa Clara, CA, USA). The resulting architecture operates on RGB images of size 80×80 or sliding windows; it is trained for the “Ships in Satellite Imagery” and by achieving frequency 270 MHz, completing the inference in 0.687 ms and consuming 5 watts, it validates the approach.

## 1. Introduction

The evolution of FPGAs with respect to the increased hardware resources and the efficiency of their programming tools has significantly affected the applications with real-time specifications. Image-processing tasks, especially those based on deep learning techniques [1] and CNNs, benefit by the utilization of FPGAs as accelerators [2]. FPGAs are advantageous for these tasks because of their ability to reconfigure and/or reprogram the architectures; consequently, the designer can follow the continuous improvement of the CNN algorithms and techniques. Among the aforementioned processes, those that are destined for edge, mobile and on-board satellite (OBC) computing have to use accelerator designs that are performance, power and resource efficient. Aiming at improving the performance of these tasks, the current paper presents a design approach for real-time image classification FPGA accelerators that can be implemented with the logic and memory resources of a single FPGA device and it shows its advantages by developing a vessel detection FPGA accelerator.

The proposed approach is effective for CNN applications with relatively low feature space [3,4,5] such as the classification problems that share similar characteristics between classes [6,7] and CNNs requiring few convolution layers, such as SAT-4/SAT-6 [8]. The proposed FPGA design approach includes three phases with each phase targeting distinct design and performance gains. The first phase introduces guidelines that lead the CNN design process with TensorFlow to a model of reduced computational and memory requirements but of high classification accuracy. In the second phase, the model is transformed into a fixed-point Bit-Accurate Model (BAM) simulating the hardware calculations and allowing the designer to decide on the arithmetic representation of the model’s parameters that provide the optimal trade-off between bit-width reduction and accuracy losses. For the third phase, we use a library of algorithm-specific blocks in Very High-Speed Integrated Circuit Hardware Description Language (VHDL) implementing the CNN functions with fixed-point arithmetic. These blocks, along with our proposed methodology for mapping the CNN to the FPGA, provide the means to the FPGA designer to initiate the third phase and implement a distinct module for each CNN layer. The completion of the third phase places these modules in a pipeline fashion forming a streamline architecture, to result in an efficient FPGA accelerator with respect to power consumption and resource utilization while saving significantly on the development time.

Considering vessel detection as the target application, we exploit the proposed approach to design an accelerator within a single FPGA device, which decides whether there is a vessel [7], in the input image. This image classification task utilizes a CNN trained for the Planet’s “Ships in Satellite Imagery” dataset [9] and the resulting FPGA accelerator using the resources of only the Xilinx Virtex 7XC7VX485T device (operating on a Xilinx VC707 board) achieves almost 98% prediction accuracy and high throughput by classifying an 80×80 RGB 24 bits/pixel image in 0.68 ms. Moreover, the accelerator can be used in a sliding window application for scenes up to 4 K. To compare the FPGA’s performance, we executed our code on the low power Intel’s Myriad2 processor (Intel Corporation, Santa Clara, CA, USA) [10] used for cameras and OBC [11,12] and the edge-computing NVIDIA’s Jetson Nano (NVIDIA Corporation, Santa Clara, CA, USA) [13] either on the Jetson’s ARM processor (Arm Ltd., Cambridge, UK) or the GPU.

The paper is organized as follows: the following section reports related results in the literature; Section 3 provides the necessary background for the target application of the paper, i.e., the vessel detection; Section 4 introduces the approach for designing the CNN and mapping on the FPGA; Section 5 describes the proposed FPGA accelerator for the vessel detection application; Section 6 details the corresponding FPGA and performance results; finally, Section 7 concludes the paper.

## 2. Related Work

Researchers have studied and provided FPGA accelerator solutions for CNNs based mainly on the automated software development tools such as the High-Level Synthesis (HLS) [14,15,16,17,18,19], due to short development time and hardware abstraction. The approach followed in [14] improves the time of the entire design process by parallelizing the CNN C code with Pthreads and by optimizing the FPGA accelerator through software/constraint changes only. The authors of [15] target feasibility at low cost by choosing inexpensive FPGA devices and cores for their accelerator. The authors of [16] focus on optimizing the accelerator’s performance by considering the architecture’s throughput combined with the external memory’s throughput. The FPGA accelerator of [17] interfaces with a host PC and it utilizes off-chip memories with the loading/storing of the intermediate results optimized for higher bandwidth. The authors of [18] report an FPGA accelerator template with an HLS FPGA architecture consisting of a cluster of Multiply Accumulate Processing Elements for convolution acceleration; this work focuses on a design flow selecting processing schedules that minimizes external memory accesses and buffer size by means of data reuse. The authors in [19] present an accelerator based on a single-processing engine that targets standard and depthwise separable convolution. In this work, the authors aim to reduce the delay added by the off-chip memory data exchange by using a data stream interface and ping-pong on-chip cache. All of the HLS design approaches, however, prevent experienced designers from optimizing the HDL code towards a more efficient FPGA architecture with respect to resource utilization, throughput and energy consumption [20]. The authors in [12] present an on-board satellite FPGA accelerator for CNN inference, which utilizes a single processing unit with external DRAM memory, developed with VHDL code. Note here that the current work focuses on streamline architectures that implement the contiguous CNN layers in a pipeline fashion and differs to the implementation of a systolic array that is reconfigured each time it computes a CNN layer [16]. Hence, the advantages of the proposed designs are to avoid idle computing and memory [21] resources, use only the on-chip (FPGA) memory and extensive pipeline, features that lead to improved resources utilization, reduced latency and power consumption [22,23].

Regarding the results related to vessel detection [24,25], which the current work has as target application, most of the published results exploit algorithmic techniques to improve the execution time. The widely known approaches are R-CNNs [26], Faster-RCNN [27], You Only Look Once (YOLO) [28] and Single Shot MultiBox Detector (SSD) [29]. Another approach in [30] recognizes the key parts of the vessel and classifies the ship’s identity by using these key parts. These classification results are then voted on for the decision of the ship’s identity, with the highest achieved accuracy being 92.63%. Hardware accelerators developed solely for vessel detection are detailed in [31] but without CNNs: they propose a technique based on statistical analysis, of the inspected and neighboring areas to distinguish the “possible ship” to other objects and using the geometric features of the target, they decide whether the target is a ship, achieving a 90% success rate. The large number of approaches, algorithmic techniques and results related to the vessel detection is due to the attention that vessel detection as a task has gained over the last two decades; the following section provides background.

## 3. Background on Vessel Detection

Vessel detection is among the most important tasks of Maritime Domain Awareness [24,25] including all the activities associated with maritime activities that could impact upon the security, safety, economy or the environment and which are related to any navigable gateway and the associated infrastructure, people, cargo and vessels. For the corresponding applications, vessel detection is the keystone because it has a very extended scope of applications in the areas of maritime safety and rescue missions, marine traffic control, sea pollution, maritime spatial planning, management of remote fisheries, area fishing control, illegal migration, customs control, observation of naval borders, etc. Referring to the ships and all floating manufactured objects as vessels, and given that it is rather straightforward to distinguish an object in optical images produced either by space, drones or harbor cameras, the processes that identify the vessel in the image frames play a key role in the above applications.

Moreover, note that for ships greater than 300 tons, it is mandatory to use shipborne transponders to report their position. Smaller ships though, do not have to own and use these devices. Ships that engage in illegal operations either turn them off or they try to deceive the authorities with false reports of their position. Hence, vessel detection can effectively support maritime domain awareness. Consequently, the exploitation of images, especially satellite imagery, plays a key role for locating vessels on the sea surface. A notable example is satellite-based radar images most often, Synthetic Aperture Radar (SAR), which are commonly used for maritime surveillance because they provide the ability to detect the vessels either in the case of both clear or cloudy skies. The interest in using optical images in the applications of maritime surveillance escalated significantly due to the availability of optical-imaging satellites.

Considering the problem definition, vessel detection can be envisaged as a task of detecting an object given that the background in most cases has the characteristics of the surface of the water. Following the latter model, the researchers and the engineers focused on providing solutions in terms of automated analytical methods for remote sensors. These efforts are the consequence of the existence of the large number of Earth-orbiting sensors and their ability to generate and transmit big volumes of data. Hence, the detection systems have to process large volumes of sensor data and in many cases to conform to near-real or real-time requirements. Accordingly, the limitations in the execution time, as well as the restrictions in the power consumption and the resource utilization, call for power and resource-efficient hardware accelerators [11]. A generic approach for the vessel detection is to receive an input image of size k×k pixels, on which it will perform the calculations of the trained CNN model, the convolution with the filters kernels, the max-pooling and finally the classification with a fully connected neural network. This operation is repeated on overlapping image patches extracted from a large image of size x×x pixels, where x>>k, gathering the patches that contain vessels and discarding the remaining.

## 4. CNN Design Approach

The current section introduces the three distinct phases of the proposed image-classification FPGA accelerator design approach. It begins by presenting the first phase with the guidelines for the CNN model design. Then, for the second phase, it describes the development of the fixed-point BAM representation of the CNN floating-point model based on the factors that play a key role in the design of the entire FPGA accelerator. Finally, the third phase introduces the configurable VHDL blocks and the mapping methodology of the CNN layers to the FPGA by utilizing these blocks. The result is a map of the CNN layers on a pipeline of modules, where each module is optimized to the corresponding layer computations. The proposed streamline architecture designs save significantly on the FPGA resources compared to the architectures that implement all the CNN layers on a systolic array [16] and leave idle resources as the layers progress.

### 4.1. CNN Design Space Exploration

This work focuses on single FPGA device solutions for image recognition applications and more specifically, binary and limited feature space classification tasks. Consequently, the design process has to consider all the factors reducing the resources’ requirements. For this purpose, in the first phase the designer uses the TensorFlow estimator API to design the CNN’s model targeting to fit within a single FPGA’s resources. Focusing on all the key factors of the images under consideration, the designer can develop the model by keeping to the following guidelines for:

- Number of layers: the neural networks for the low-feature-space image-classification applications can achieve a high accuracy rating even with a relatively small number of convolution layers [8].
- Size of convolution kernels: considering the images that are relatively small, the recognized objects tend to occupy a large portion of the image and hence, large and medium-size convolution kernels suffice.
- Choosing the size of the pooling layers windows: the feature space is relatively limited and hence, the use of 4×4 pooling layers will not affect the accuracy, though it will significantly improve the resources’ requirements of the succeeding layers.
- Padding avoidance: this is used throughout the CNN because: (a) it does not affect the accuracy and (b) given that the majority of the target applications have objects located at or close to the image center, we do not need to preserve the size of the feature maps.
- Divisibility: it refers to the divisibility of each convolution layer’s output size by the kernel size of the succeeding pooling layer. If it is applied, it will: (a) allow the omission of padding with no accuracy loss and (b) lead to an efficient pipeline for the contiguous layers.

### 4.2. Bit-Accurate Model Development

During the second phase, the designer develops the BAM of the designed and trained image classification CNN. The BAM emulates the exact same fixed-point calculations that the hardware accelerator performs. For the BAM, we perform quantization of the CNN model’s trainable parameters, starting from the 32-bit floating point representation provided by TensorFlow to a desired Qm.n fixed-point representation. The number of bits for the integer part *m* and fractional part *n* are accepted as input parameters to the BAM. This allows the designer to perform a trade-off study between saving on FPGA resources due to the reduced bit width of the CNN parameters and maintaining high classification accuracy as a result of the reduced arithmetic precision.

### 4.3. VHDL Blocks

The proposed approach combines the VHDL advantages with an efficient, with respect to the development time, design methodology for CNN accelerators. Multiple instances of configurable and reusable VHDL blocks, each with different configurations, are used for the development of each layer. The following subsections present the reusable VHDL blocks developed in this work.

#### 4.3.1. Input Block

**Figure 1:**
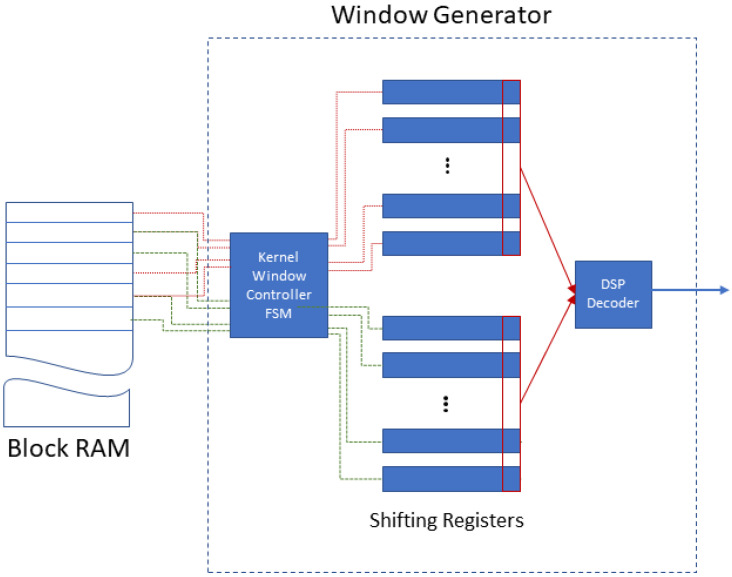
Input block architecture.

This block consists of a *Block RAM* that stores one RGB channel of a full image and a *Window Generator* as shown in Figure 1. The *Window Generator* formulates the input to the following convolution layer as windows of size equal to the convolution layer’s n×n kernel (e.g., 3 × 3, 5 × 5, etc.). It uses *n* shift registers with each register containing one image row, in order to avoid the indexing of pixels and thus, lead to improved performance. The *Kernel Window Controller FSM* of the *Window Generator* reads *n* rows from the *Block RAM* and copies them into the first set of *n* *Shifting Registers*. The *DSP Decoder* formulates the n×n window: the first *n* pixels (memory words) of each of the *n* *Shifting Register*, are routed in parallel to the input of the following convolution layer. To create the next window, we shift the *n* registers by one pixel. There are two sets of *Shifting Registers* forming a double input buffer. If the following convolution layer uses n×n kernels, the *n* shift registers forward an input n×n window per cycle to fully pipeline the two layers. The following parameters are configurable: (a) image size, (b) the *n* registers, (c) the kernel n×n and (d) pixel bit-depth.

#### 4.3.2. Convolution Block

**Figure 2:**
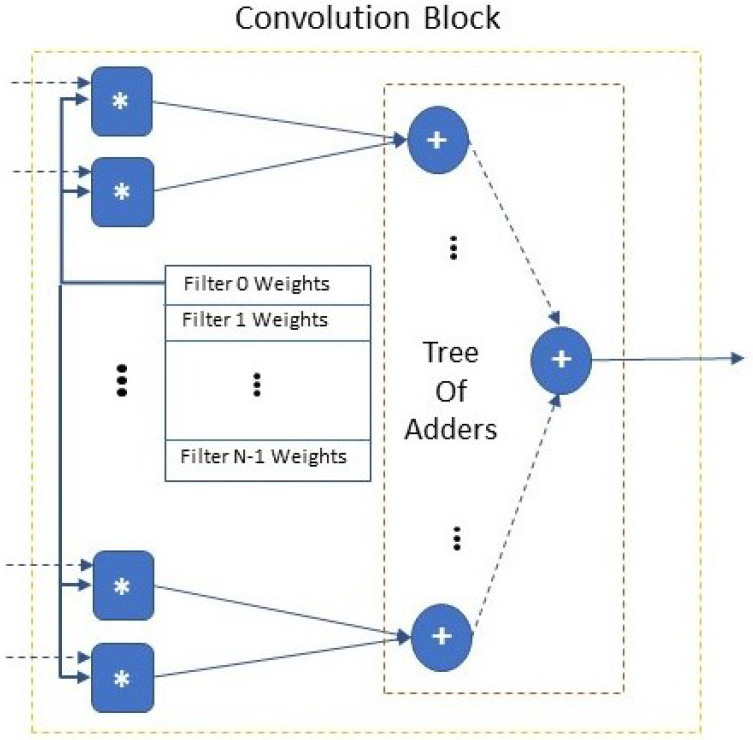
Convolution block architecture (∗ refers to fixed-point integer multiplication, + refers to fixed-point integer addition).

The *Convolution Block* (Figure 2) receives a single channel of the input image (or a single feature map) in the format of kernel-sized windows (n×n) and it calculates the convolution of a single filter’s kernel with the input. The *Convolution Block* includes n×n multipliers; each multiplier has input one pixel of the n×n window and the corresponding kernel weight. Different filter kernels are stored at the on-chip ROM of the *Convolution Block*. To calculate the output of the *Convolution Block*, a tree of adders (of height $\left\lceil log_{2}\left(n\times n\right)\right\rceil$) [completes the addition of all the products of the multipliers sum the product of each multiplier in a pipeline fashion.

#### 4.3.3. Pooling Block

**Figure 3:**
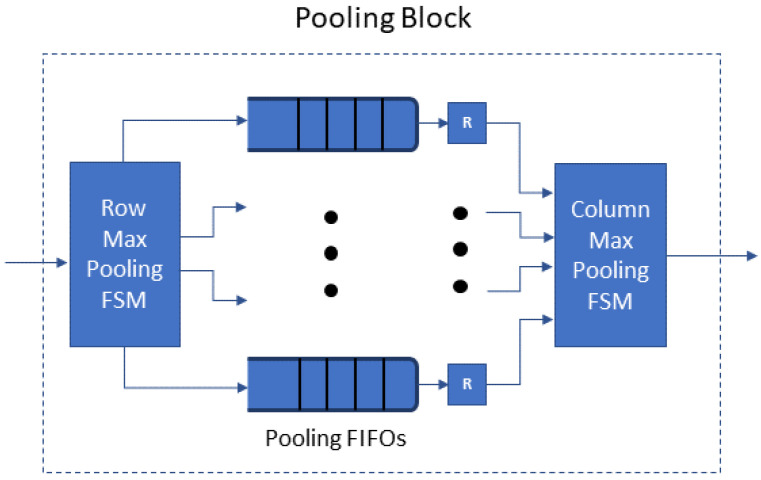
Pooling block architecture.

The *Pooling Block* (Figure 3) receives the feature map produced by a preceding convolution layer: a k×k array forwarded one value at each cycle. The *Pooling Block* selects the maximum value of each l×l window, for all the windows in the feature map with stride *l* (e.g., 2×2 or 4×4 max pooling) and outputs the k/l×k/l array of the above maximum values. In detail: first, from the k×k array, the sub-block *Row Max Pooling FSM* obtains the maximum of each *l*-tuple of values of each row to provide a k×k/l array; *l* registers are written in *l* consecutive cycles and we choose the maximum of the *l* registers. There are *l* *Pooling FIFOs*: the *Row Max Pooling FSM* stores the result in the next available FIFO and marks it as the active *Pooling FIFO*, i.e., the k/l results of the rows 0l, 1l, 2l, etc., will be stored in the first *Pooling FIFO*, those of the rows 0l+1, 1l+1, 2l+1, etc., in the second and so on. When *l* rows of the output feature map (l×k/l values) are stored at the *Pooling FIFOs*, the *Column Max Pooling FSM* starts the vertical max pooling; it chooses the maximum of *l* data (one from each *Pooling FIFO*) to produce the k/l×k/l array.

#### 4.3.4. Vector Multiplier

The *Vector Multiplier* realizes a fully connected Layer neuron; it computes the dot product of the 1-D input vector *I* (the flattened result of the preceding layer), which is received one point at a time, with the corresponding row of the fully connected layer’s weight matrix. The weight matrix *W* is stored in a ROM, where each memory word contains the weight of every neuron for each input. At each cycle, the input value of *I* and the corresponding row of *W* are multiplied and the block accumulates the result, which is forwarded to the following blocks.

#### 4.3.5. ReLU and Output Block

The *ReLU Block* is a 2-to-1 multiplexer. The select bit of the multiplexer is the Most Significant Bit (MSB) of the input value. If the MSB/select is “1”, the input is a negative number and the multiplexer outputs zero, otherwise it forwards the input to the output.

**Figure 4:**
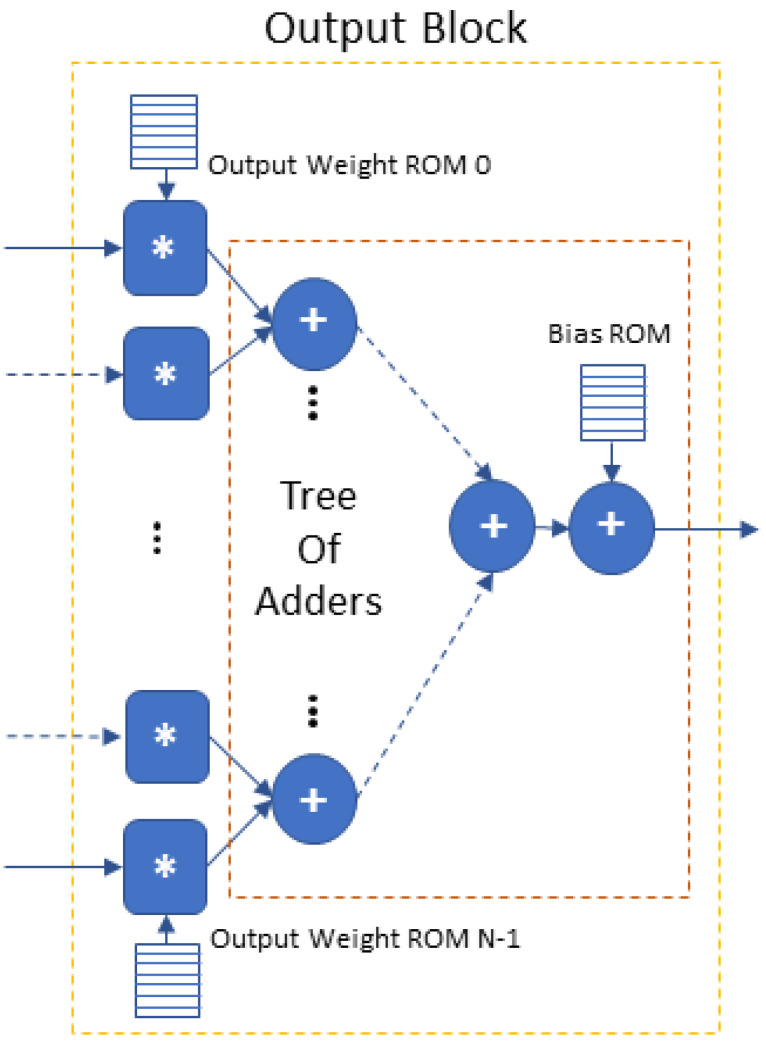
Output block architecture (∗ refers to fixed-point integer multiplication, + refers to fixed-point integer addition).

The *Output Block* is the CNN’s final fully connected layer. Its architecture is shown in Figure 4. It executes the matrix multiplication of the flattened input array *I* with the weights *W* of the output neurons and then adds the bias. In a pipeline fashion, it is executed once for each output neuron/class.

### 4.4. Methodology for Mapping the CNN on the FPGA

The current section describes the major considerations and recommendations for mapping the CNN functionality on a VHDL architecture by utilizing the above blocks.

The proposed approach uses the mapping to result in a streamline architecture that implements all the layers of the CNN as a pipeline of modules: each module implements a CNN layer’s computations. This allows flexibility in the parallelization strategy of the computations of each layer (implemented as a module); our proposed approach aims at parallelizing the layers in a way that enables extensive pipelining between them and minimizes the use of intermediate buffers. In more detail, for the acceleration of binary and limited feature space classification tasks with shallow CNNs that this approach targets, the streamline architectures and the proposed design approach have the following benefits:

(a) High efficiency in resource utilization and computing since all hardware is generated specifically for each CNN layer (module) and the layers are pipelined.
(b) Significantly reduced memory requirements for the intermediate results and use of buffers only on the on-chip memory. The extensive pipeline of the proposed approach allows for succeeding layers (modules) to directly consume the data generated by the preceding ones and thus minimize the buffering of the intermediate results.
(c) Reduced latency for shallow CNNs designed for the target limited feature space classification tasks. This is achieved by the parallelization strategy, the pipelining between the VHDL implemented layers (modules) and the use of only low-latency on-chip RAM.

The resource utilization and power-efficient design approach has to focus on the following characteristics. The key issue is to keep the memory and DSP requirements of the CNN accelerator design within the limits of the target FPGA device. Consequently, the objectives of CNN accelerator’s design are first, the minimization of the buffering between consecutive layers; second, the required memory of each layer; and third, the real-time performance of the accelerator. The methods for improving the key issues of the FPGA accelerator are:

- Buffers between layers and speed-up: The effort is given to parallelize the *N* filters in each convolution layer (except the first). Assuming that a convolution layer is designed with *N* filters, then the accelerator can have *K* parallel *Convolution Blocks* to complete the *N* convolution filters in N/K steps. The accelerator design with K=N is preferable because first, it maximizes the speed-up; second, it allows the pipelining of the input to every convolution block and avoids the buffer between this and its preceding layer.
- Reduce the memory of each layer: each convolution layer produces *N* feature maps and apart the first accumulates these in *N* memories. The size of each of these *N* memories depends on the size and the number of the preceding pooling layers. We denote by ${\left(sp_{i}\right)}^{2}$ the dimensions of the (i+1)th pooling layer. If the input image has size Q×Q and there are *p* pooling layers of sizes $sp_{0}\times sp_{0},\;sp_{1}\times sp_{1},\;\ldots ,\;sp_{p-1}\times sp_{p-1}$ each memory (of the *N* memories of the current layer) has size $\left[Q\times Q\right]/\prod_{i=0}^{i=p-1}\left(sp_{i}\times sp_{i}\right)$. Hence, higher-dimension pooling layers reduce the memory size and allow us to implement *N* parallel filters with their individual memories.
- The First Convolution Layer. The proposed parallelization technique for this layer leads to the balance of the speed-up against the available number of DSP blocks and block RAMs of the target FPGA device. The key computational role is realized by a parallel *Structure* consisting of one convolution block per channel; these blocks compute the convolution of all the input image’s channels (3 channels and 3 corresponding blocks in the case of RGB). Each block completes the convolution in real time and it forwards each result to the following pooling layer without a buffer, a design feature that significantly improves the memory requirements since the first convolution layer operates on the full-size input image (without any downsampling). The use of one (1) *Structure* to complete all the filters of the first convolution layer is resource efficient. It creates a parallel *Structure* by using first, 3 Convolution Blocks to compute the convolution of all the input image’s channels (3 in the case of RGB) and second, a tree of adders adding each channel’s convolution result in real time, forwarding it to the following pooling layer without a buffer. Avoiding this buffer is advantageous because it significantly improves the memory requirements considering that the first convolution layer operates on the input image without any downsampling. A single parallel *Structure* has to repeat this operation for every filter of the first convolution layer, but is the most resource efficient. If the following pooling layer has size $sp_{0}\times sp_{0}$, this layer will be slower by ${\left(sp_{0}\right)}^{2}$. Depending on the target FPGA’s resources, we can use *k* instances ($k\leq {\left(sp_{0}\right)}^{2}$, where ${\left(sp_{0}\right)}^{2}$ the dimensions of the first pooling layer) of this *Structure* in parallel to improve the speed-up by *k*. We note here that each additional parallel *Structure*: adds a set of 3 convolution blocks, increasing the use of the FPGA DSP blocks; adds another memory buffer at the interface between the first pooling layer and the second input layer. However, the size of each additional input buffer is considerably reduced due to the high dimensions of the first pooling layer. Using *k* such *Structures* and the *k* buffers is limited by the available DSP blocks.
- Scalability. The aforementioned techniques lead to a scalable FPGA accelerator design. The architecture of the first convolution layer enables the engineer to opt for more performance or optimize the design for FPGA devices with limited resources. Moreover, the fully connected layers can use a *Vector Multiplier* per neuron: parallelizing the neurons is advantageous leading to a layer design irrespective of the size and the number of the feature maps produced by the preceding layer; more importantly, it is scalable.

## 5. Vessel Detection CNN FPGA Accelerator

The following paragraphs employ the proposed design approach to develop a vessel detection FPGA accelerator that can also be used in sliding window applications of large images. The use of the proposed vessel detection FPGA accelerator can be realized in the context of an FPGA system, in which the accelerator interfaced with a host processor/FPGA-engine and is receiving windows of 80 × 80 for classification of a larger image stored in central mass memory (e.g., 3081× 1597 in Planet’s dataset used) obtained from the camera sensor.

### 5.1. Model Architecture and Training

The model was trained with the “Ships in Satellite Imagery” Kaggle dataset. It contains 4000 80 × 80 RGB images in total, labeled with either “ship” or “no-ship” binary classification: 3K images were selected for the training process and the remaining for model validation.

**Figure 5:**
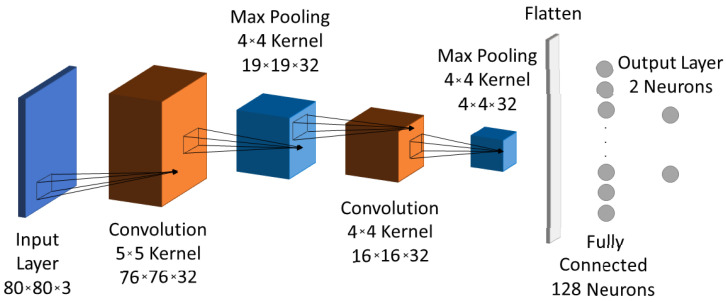
Model architecture.

A variety of training processes was performed with the TensorFlow Estimator API in Python to create a CNN model close to optimal with respect to prediction accuracy, number of operations and resources requirements. The CNN design space exploration (described in Section 4.1) resulted in the final model architecture shown in Figure 5. The CNN model consists of 84K weights optimized using the Adam optimizer with the cross-entropy loss function; it achieves 97.6% accuracy after 50 epochs. It compares favorably to similarly trained models due to the following results of the design study:

- Number of convolution layers: The proposed CNN with only two convolution layers achieves an accuracy of 97.6%, which is close to that of CNNs with more, e.g., a CNN with three convolution layers before any of the proposed optimizations achieved 98.5% accuracy.
- Ship orientation: The ship orientation is limited and, along with the proportion of the 80 × 80 image that the ship occupies, it leads to the use of 32 filters per convolution layer for achieving the best accuracy–computational cost trade-off.
- Max pooling layer: size 4×4 achieved accuracy similar to that of size 2×2.
- The kernel’s size for each convolution layer: the first achieved improved accuracy with a 5×5 kernel, while the choice for the second convolution layer is a 4×4 kernel because its output has to be divisible by the following max pooling layer. As a result we did not use padding in the convolutions since this does not induce accuracy loss.
- Fully connected layer’s neurons: 128 neurons of the fully connected layer is the minimum number to use in order to avoid prediction accuracy loss.

### 5.2. Bit-Accurate Model (BAM)

The design flow, following the realization of the TensorFlow model for vessel detection, develops a bit-accurate model (BAM) that represents the exact operations and calculations in integer arithmetic that takes place in the FPGA. We note here that the input image is represented in RGB with 8-bits per pixel and the BAM keeps (does not reduce), for each pixel, the input bit-depth. Each parameter of the CNN model (weights, biases) is represented as a fixed-point number with 1-bit for the sign, 1-bit integer part and 6-bit fractional parts (Q2.6). Throughout the BAM, we preserved the 6-bit fractional part by truncating the result of each multiplication. In order to avoid accuracy losses due to the overflow after consecutive additions, the integer part is increased and the final results are represented in Q11.6.

### 5.3. FPGA Accelerator

**Figure 6:**
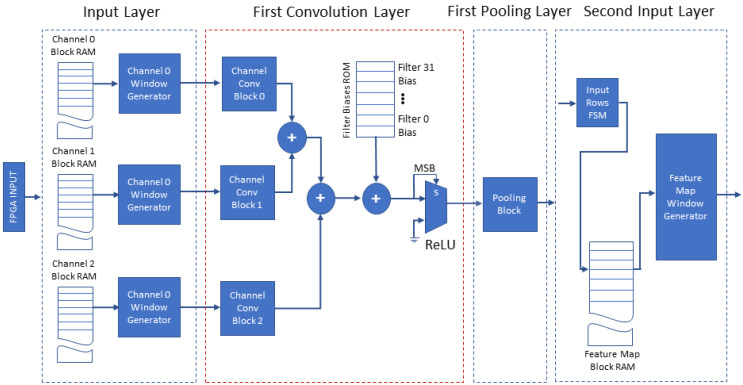
FPGA Architecture of the input layer, first convolution and pooling layers and the second input layer (+ refers to fixed-point integer addition).

**Figure 7:**
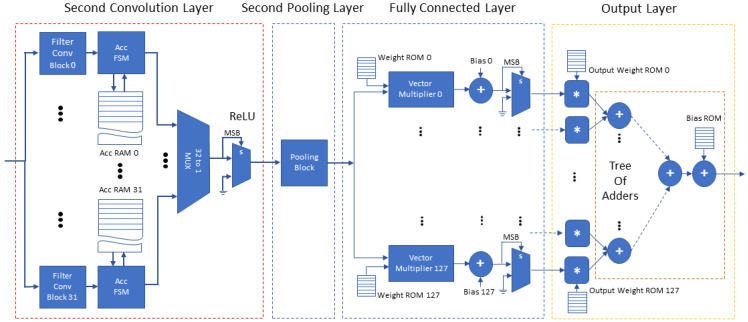
FPGA architecture of the second convolution and pooling layers, fully connected layer and output layer (∗ refers to fixed-point integer multiplication, s refers to the “select” input pin of the multiplexer, + refers to fixed-point integer addition).

The proposed accelerator’s architecture consists of eight structural blocks on which we map the functionality of the software model blocks: (a) the input layer, (b) the first convolution layer, (c) the first pooling layer, (d) the second input layer, (e) the second convolution layer, (f) the second pooling layer, (g) the fully connected layer and (h) the output layer. The overall architecture is illustrated in two figures, Figure 6 and Figure 7. The proposed accelerator exploits the parallelization of the CNN model in order to increase performance, minimize buffering and improve the throughput via pipelining of its operations. The following paragraphs present the significant details of the structure and operation of the proposed accelerator’s blocks and their advantages.

Figure 6 depicts the four leading blocks of the architecture (input layer, first convolution layer, first pooling layer and second input layer). The architecture uses the blocks described in Section 4.3: the input layer with the *Window Generators* and the first convolution layer including three *Convolution Blocks*. Their output is forwarded to the ReLU and the first pooling layer consisting of one pooling block configured for 4×4 max pooling. The second input layer includes a single input block. This design minimizes the memory required by the proposed accelerator in two ways. The first convolution layer calculates and adds in parallel the convolution of each input image channel with the corresponding kernel producing one complete output feature map, pipelining each value to the first pooling layer without buffer use. The calculations are repeated for the remaining 31 feature maps, with the corresponding filter kernels. The second convolution layer calculates the 32 filter convolutions on each received feature map in parallel and buffers the 32 results for accumulation. The required buffering at the output of this layer is reduced to 32 arrays of 16×16 13-bit values, because at this stage we have already executed the first pooling layer (4×4 max pooling). The latter shows the advantage of the proposed approach when it is used for shallow CNNs, because considering a systolic array accelerator for the same task, it would require a total of 2.03 Mbit to store the intermediate result of the output of the first convolution layer. In contrast, the proposed streamline architecture uses the buffering of intermediate results only at the end of the second convolution layer, following the downscale of the data by previous pooling operations: this is only 106.50 Kbit and hence, it achieves a 19.1× reduction in the required memory.

Another key element of the proposed accelerator’s architecture is the input layer, which is shown in Figure 6; its design is based on the FPGA’s features. The FPGA can support a variety of interfacing methods with the host such as PCIe, Ethernet and USB to receive the image. The input layer stores each channel (RGB) of the input image row by row in the corresponding *Channel Block RAM* (on-chip memory), so that we can read a whole row in a single clock cycle. These blocks, along with the three *Window Generators* of the three *Input Blocks*, constitute the input layer. The *Window Generators* are configured to accept one 80×80 image (one image channel each) and generate all the windows of size 5×5 of that image channel; they operate as described in Section 4.3.1. When the image is stored in each *Channel Block RAM*, the three *Window Generator* blocks operating in parallel load the three distinct channel windows of size 5×5 in parallel to the three corresponding *Channel Convolution Blocks* of the first convolution layer, as shown in Figure 6. Three distinct RGB windows of size 5×5 forwarded in parallel at each clock cycle to the *Convolution Blocks* in a fully pipelined operation.

Figure 7 depicts the second half of the proposed accelerator’s architecture (the second convolution and pooling layers, the fully connected layer and the final output layer). The second convolution layer includes 32 *Filter Convolution Blocks*, each block configured for 4×4 convolution kernels. The second convolution layer receives, one by one, the feature maps of the previous layers and performs the 32 filter convolutions of this layer in parallel with 32 *Filter Convolution Blocks*, each of which accumulates the result in a dedicated *Accumulator RAM* of size 16×16 words. Each *Filter Convolution Block* stores the kernel weights associated with each input feature map in an internal Block RAM. The results of this layer are complete when every feature map of the previous layer is received and processed. At the final accumulation step, each filter’s bias is added and the *Accumulator RAM* contents of each *Filter Convolution Block* are forwarded, in a continuous stream (in filter order) to the second pooling layer. The second pooling layer is similar to the first pooling layer, also configured for 4×4 pooling, where memories act as a buffer in order to provide an uninterrupted flow of data to the succeeding fully connected layer. Finally, the fully connected layer uses 128 parallel *Vector Multipliers*, one for each neuron. When all the multiply–accumulate steps are complete, the 128 parallel multipliers and a tree of adders calculate the inference result.

Although the CNN vessel accelerator improves the performance of CPU, GPU and edge processors, as is shown in Section 6.2, it is worth noting that the entire CNN vessel accelerator architecture can be configured to operate on two distinct input frames in a pipeline fashion. In that configuration, while the first frame processing will occupy the fully connected layer, the two convolutional layers will be dedicated to the process of the second (following the first) frame.

## 6. Vessel Detection FPGA Accelerator Results and Comparison

This section presents the results of the proposed accelerator’s implementation on the Xilinx VC707 board and a comparison with the corresponding performance of our code executed on: (a) the low power Intel’s Myriad2 processor, (b) the edge-computing NVIDIA’s Jetson Nano Jetson’s ARM processor and (c) the Jetson Nano GPU.

### 6.1. FPGA Implementation Results

**Figure 8:**
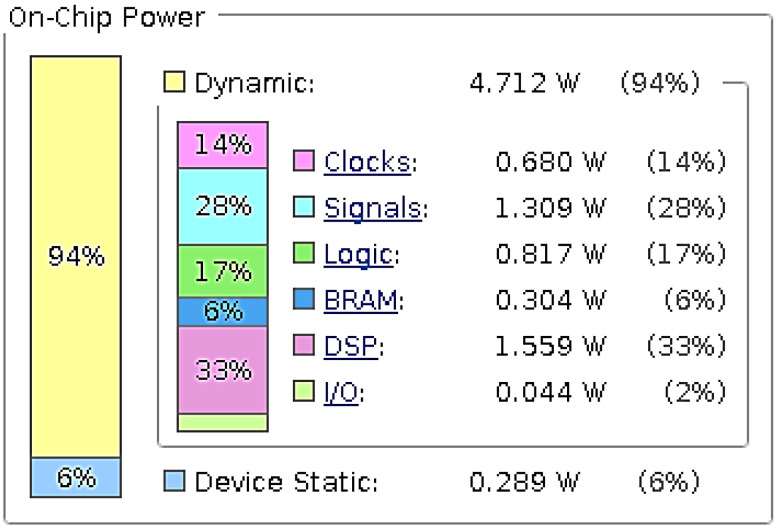
Power utilization.

The development and validation of the proposed accelerator targeted the Xilinx Virtex 7 Development board (XC7VX485T) with the use of the Vivado development tool. The resource utilization of the FPGA on the Virtex 7 board is presented in Table 1. More specifically, the proposed accelerator uses 9.37% of the FPGA’s BRAMs and 30.11% of the available DSP blocks of the FPGA device. The proposed accelerator’s power requirements are 5.001 W reported by the Vivado power estimator. Figure 8 presents the on-chip power utilization per resource type.

The FPGA implementation of the proposed accelerator has achieved a maximum operating frequency ($f_{max}$) of 270 MHz. The number of operations per second of the accelerator is 52.8 GOP/s and the processing time for a single input image (or a 80×80 sliding window) is 0.687 ms. In order to showcase an indicative baseline evaluation result, Table 2 presents the execution time comparison of the proposed accelerator to the CPU and GPU software implementations. The CPU and GPU software implementations are based on the TensorFlow implementation of the model executed with a single image as input and targeting the Intel(R) Core(TM) i7-9700K CPU @ 3.60 GHz and the NVIDIA GeForce RTX 3080 correspondingly. The CPU processes a single input image in 4.696 ms while the GPU processes the same input image in 2.202 ms. The proposed accelerator achieves a speed-up of 6.836 and 3.205 when compared to the CPU and GPU correspondingly.

### 6.2. Comparison to Edge Devices and Low Power Processors

In order to evaluate the proposed approach, we compared the performance of the vessel detection CNN FPGA accelerator to the other edge devices, which have high performance at low-power consumption according to their specs. Notable representatives are the NVIDIA’s Jetson Nano and the Intel’s Myriad2 processor. The Jetson Nano of 472 GFLOPS (FP16) at 10 W includes an ARM processor and an 128-core Maxwell GPU targeting computer vision and deep learning applications. The Myriad2 processor is being utilized for on-board satellite computing applications in missions [32] and research projects [11] due to the fact that it has undergone extensive radiation characterization [33] in order to be deemed suitable for space applications. It has two Leon and 12 SHAVE processors and it is optimized for machine learning applications, which can aggregate 1000 GFLOPS (FP16) with at most 1W consumption. Moreover, it includes a multicore on-chip memory subsystem (2 MB), called the connection matrix (CMX) memory and low-power DDR3 DRAM (512 MB).

The comparison is based on a sequential C code for vessel detection. This was executed on a single core of the Jetson’s Nano ARM CPU and measured at 440 ms. From this point, we developed a custom CUDA-accelerated application taking advantage of the 128 CUDA cores. The mapping of calculations to the grids of thread blocks optimizes the scheduling of warps on the 128 CUDA cores. The shared memory is used to store global data in a thread block and the intermediate results. The execution time of the CUDA application is 20.3 ms.

The development on the Myriad2 starts with the optimization of the sequential C code, using the CMX, DDR and cache memories efficiently; this single core application took 56.27 ms with less than 0.5 W. The parallel Myriad2 code uses the 12 SHAVES, by dividing the CMX memory between them, minimizing the required memory of each SHAVE by pipelining the operations of each processor, the parallel code takes 14.6 ms at 1 W.

The detailed results are presented in Table 3. The proposed FPGA accelerator achieves the highest performance, regarding execution time and median power consumption; however, the highest performance per Watt was found among the other two devices. The Myriad2 is the most power efficient by consuming 1 W, while its performance is one order of magnitude lower than the FPGA accelerator. The Jetson Nano falls short in either metric with a consumption of 10 W and execution time in the same order of Myriad2, but it provides the most developer-friendly platform, which is an advantage leading to short development time and effort. The proposed FPGA accelerator has the highest performance per Watt, followed by the Myriad2 and in the last place is the Jetson Nano.

### 6.3. Comparison to Other FPGA Accelerators

This subsection aims to provide more context to the proposed approach by showcasing where the proposed accelerator stands in the field of FPGA accelerators in the literature. A straightforward comparison of the resulting accelerator to FPGA-based CNN accelerators is a challenging task [16] because:

(a) The same metrics between different FPGA accelerators may not be suitable for direct comparisons due to different FPGA platforms, benchmarking methodologies, etc.
(b) While the majority of related works focus on accelerators for well-known CNN models, this work proposes a design approach that includes guidelines for designing CNN models from scratch, resulting in a custom model for vessel detection application.
(c) This work focuses on accelerator designs for shallow CNNs suitable for binary and low feature space classification tasks while most works in the literature study complex and larger CNN models and result in substantially different architectures.

Regarding these architectural differences, the proposed streamline architectures in this work use contiguous modules for each layer of the CNN in a pipeline fashion. These architectures have particular benefits for our target applications (described in Section 4.4) and are further highlighted in the comparisons with other FPGA-based accelerators and the corresponding analysis below.

Taking into account the aforementioned considerations, Table 4 presents notable works on FPGA-based CNN accelerators, their most important features and the corresponding metric results. Note that the proposed accelerator achieves the highest operating frequency of 270 MHz; this advantage is due to the custom VHDL design of the proposed approach, especially when compared to the 100 MHz of the HLS generated designs of [16,19]. Moreover, the advantage of the streamline architecture as well as the utilization of only the on-chip memory is observed when compared to the 156 MHz of the single processing unit VHDL design of [12]. Regarding the performance, the accelerator of [16], targeting a much larger CNN model, exhibits a slightly larger performance of 61.62 GOP/s compared to the 52.80 GOP/s of the proposed accelerator; however, considering that both use the same FPGA device, the current work achieves this performance by utilizing only 843 DSPs, compared to the 2240 DSPs of [16] and hence, it results in a significantly higher DSP efficiency of 0.062 GOP/s/DSP. The reason for this improvement in hardware efficiency is the proposed mapping methodology that produces a streamline architecture with multiple layers operating at the same time with extensive pipelining, in contrast to the systolic array architecture implementing a single layer at a time [16]. Considering power consumption, the authors of [12] report 3.4 Watts while our proposed accelerator consumes 5.001 Watts; however, in that work there is no report of several features of the design that play a role in power consumption, such as CNN size and performance. The accelerator in [19] reports power consumption of 4.083 Watts but achieves lower performance per Watt compared to the proposed accelerator. Finally, the power measurements in [16] follow a different methodology by measuring the power consumption of the entire FPGA board rather than on-chip power consumption that we report and thus their measurement is not suitable for direct comparisons.

## 7. Conclusions

The current paper presented a design approach for FPGA accelerators for image classification CNNs with limited feature space targeting the edge, mobile and on-board satellite computing applications. The objective of this work was to achieve real-time performance by placing all the inference task computations and memory within a single FPGA device. The benefits of the resulting architecture are the low-power consumption, the higher operating frequency and the improved resources utilization. These advantages are shown by the FPGA accelerator for vessel detection that compared favorably to the performance of notable edge and low-power processors. Finally, the benefit of introducing the approach for the image classification on a single FPGA device, whenever this is feasible, can be shown by the vessel detection accelerator performance and compared to the optimized FPGA CNN accelerators and also to low cost ones.

## Figures and Tables

**Table 1:** Resource utilization.

Resource	Utilization	Utilization %
LUT	50,743	16.71
LUTRAM	4228	3.23
FF	70,786	11.66
BRAM	96.5	9.37
DSP	843	30.11

**Table 2:** Performance comparison to CPU and GPU.

	Execution Time (ms)	FPGA Speed-Up
FPGA	0.687	-
CPU	4.696	6.836
GPU	2.202	3.205

**Table 3:** Performance and power comparison to edge devices.

	Execution Time (ms)	Speed-Up	Power (W)
Jetson Nano CPU	440	-	10
Jetson Nano GPU	20.3	21.7	10
Myriad2 1 SHAVE	56.27	7.8	0.5
Myriad2 12 SHAVE	14.59	30.1	1
FPGA Accelerator	0.687	640.5	5

**Table 4:** Reporting the features of related results.

	[16]	[19]	[12]	Proposed Accelerator
Precision	fl. point	fl. point	fixed-point	fixed-point
	32 bits	32 bits	16 bits	17 bits
Frequency (MHz)	100	100	156	270
FPGA	Xilinx Virtex	Xilinx Zynq	Xilinx Zynq	Xilinx Virtex
	VC707	7100	ZCU106	VC707
CNN Size	1.33 GFLOP	N/A	N/A	18.122 MMAC
Performance (GOP/s)	61.62	17.11	N/A	52.80
Power (Watt)	18.61	4.083	3.4	5.001
Perf./Watt	3.31	4.19	N/A	10.56
(GOP/s/Watt)				
DSPs	2240	1926	1175	843
DSP Efficiency	0.027	0.008	N/A	0.062
(GOP/s/DSP)				

## Data Availability

The Planet’s “Ship in Satellite Imagery” dataset used for the vessel detection CNN FPGA Accelerator can be accessed via Kaggle through the following link https://www.kaggle.com/rhammell/ships-in-satellite-imagery (accessed on 13 April 2022).

## Abbreviations

The following abbreviations are used in this manuscript:

API	Application Programming Interface
BAM	Bit-accurate Model
BRAM	Block Random Access Memory
CMX	Connection Matrix
CNN	Convolutional Neural Network
CPU	Central Processing Unit
CUDA	Compute Unified Device Architecture
DDR	Double Data Rate
DRAM	Dynamic Random Access Memory
DSP	Digital Signal Processor
FF	Flip-Flop
FIFO	First-In First-Out
FPGA	Field-programmable Gate Array
FSM	Finite-State Machine
GFLOPS	Giga Floating Point Operations Per Second
GOPS	Giga Operations Per Second
GPU	Graphics Processing Unit
GTX	Giga Texel Shader
HLS	High-level Synthesis
LUT	Lookup Table
LUTRAM	Lookup Table Random Access Memory
ML	Machine Learning
OBC	On-board Computing
PCIe	Peripheral Component Interconnect Express
RAM	Random Access Memory
R-CNN	Region-based CNN
ReLU	Rectified Linear Unit
RGB	Red Green Blue
ROM	Read-Only Memory
SHAVE	Streaming Hybrid Architecture Vector Engine
SSD	Single Shot MultiBox Detector
USB	Universal Serial Bus
VHDL	VHSIC Hardware Description Language
VHSIC	Very High Speed Integrated Circuit
YOLO	You Only Look Once
